# The Interaction of NO and H_2_S in Boar Spermatozoa under Oxidative Stress

**DOI:** 10.3390/ani12050602

**Published:** 2022-02-28

**Authors:** Martin Kadlec, Eliana Pintus, José Luis Ros-Santaella

**Affiliations:** Department of Veterinary Sciences, Faculty of Agrobiology, Food and Natural Resources, Czech University of Life Sciences Prague, Kamýcká 129, 165 00 Prague, Czech Republic; pintus@af.czu.cz (E.P.); ros-santaella@ftz.czu.cz (J.L.R.-S.)

**Keywords:** gasotransmitter interaction, hydrogen sulfide, nitric oxide, oxidative stress, boar spermatozoa

## Abstract

**Simple Summary:**

The most recent experiments performed on somatic cells describe the interaction of nitric oxide (NO) and hydrogen sulfide (H_2_S) on various levels. In male gametes, these two gasotransmitters have been studied individually up until today. Both NO and H_2_S participate in crucial sperm structural and functional changes before and after ejaculation. Moreover, the two gasotransmitters can augment or mitigate the negative impact of oxidative stress, depending on the concentration. Oxidative stress is a concomitant condition to various male reproduction disorders. In this experiment, we investigated in vitro the simultaneous application of NO and H_2_S donors, which was compared to single-donor application (NO or H_2_S) at 100 nM concentrations in boar spermatozoa under oxidative stress. The evaluation of sperm qualitative traits revealed a positive effect of the combination of the two donors in DD treatment on progressive motility and plasma membrane integrity compared to the control sample under oxidative stress (CtrOX). The results of this experiment indicate that the combination of NO and H_2_S donors exceeds the effect of single-donor application under given conditions. In conclusion, our research indicates the importance of gasotransmitter interaction in male gametes.

**Abstract:**

Various recent studies dedicated to the role of nitric oxide (NO) and hydrogen sulfide (H_2_S) in somatic cells provide evidence for an interaction of the two gasotransmitters. In the case of male gametes, only the action of a single donor of each gasotransmitter has been investigated up until today. It has been demonstrated that, at low concentrations, both gasotransmitters alone exert a positive effect on sperm quality parameters. Moreover, the activity of gaseous cellular messengers may be affected by the presence of oxidative stress, an underlying condition of several male reproductive disorders. In this study, we explored the effect of the combination of two donors SNP and NaHS (NO and H_2_S donors, respectively) on boar spermatozoa under oxidative stress. We applied NaHS, SNP, and their combination (DD) at 100 nM concentration in boar spermatozoa samples treated with Fe^2+^/ascorbate system. After 90 min of incubation at 38 °C, we have observed that progressive motility (PMot) and plasma membrane integrity (PMI) were improved (*p* < 0.05) in DD treatment compared to the Ctr sample under oxidative stress (CtrOX). Moreover, the PMot of DD treatment was higher (*p* < 0.05) than that of NaHS. Similar to NaHS, SNP treatment did not overcome the PMot and PMI of CtrOX. In conclusion, for the first time, we provide evidence that the combination of SNP and NaHS surmounts the effect of single-donor application in terms of PMot and PMI in porcine spermatozoa under oxidative stress.

## 1. Introduction

The importance of nitric oxide (NO) and hydrogen sulfide (H_2_S) in male reproduction has been widely recognized [1,2,3,4,5]. Together with carbon monoxide (CO), the members of the gasotransmitter family participate not only in spermatogenesis, but also in epididymal sperm maturation [6,7]. The CO, NO, and H_2_S represent the main widely recognized signaling gaseous molecules [8], although other potential gasotransmitters are emerging (e.g., ammonia, methane, or hydrogen) [9]. Unlike the other gasotransmitters, the role of NO in sperm maturation is more controversial, as the elevated concentrations of NO resulting from abundant pathological conditions (e.g., inflammation or varicocele) are connected with decreased semen quality [10,11]. Both gasotransmitters, NO and H_2_S, participate in crucial sperm changes that occur prior to fertilization [6]. Apart from tyrosine phosphorylation, the NO is known to activate sGC, which produces cGMP, and thus increases sperm motility [6]. Moreover, it can also protect membrane from lipid peroxidation by increasing the ratio to O_2_^−^ [12]. In the case of H_2_S, the activation of mitogen-activated protein kinases (MAPK) [13], PI3K/Akt pathway [14], and mitochondria [15] is associated with increased sperm motility. Moreover, H_2_S is known to increase antioxidant capacity [4] through activation of superoxide dismutase (SOD) [15]. Regarding both gasotransmitters (NO and H_2_S), the effect is determined by the type of donor and its concentration that is used. Additionally, the effect can also be affected by the presence of other reactive species (RS), which may result in the formation of more reactive substances or substances with increased signaling potential [16,17]. Therefore, RS are carefully controlled by cellular mechanisms, preventing overaccumulation and oxidative stress [18].

Oxidative stress is an underlying concomitant condition to several male reproductive disorders, in which high levels of reactive species cause sperm dysfunction (e.g., decreased sperm motility, impaired membrane and DNA integrity, increased lipid peroxidation, and infertility [19]. Particularly, sperm cells are highly sensitive to supraphysiological levels of RS due to the high content of polyunsaturated fatty acids in their membranes and their limited antioxidant defense [20]. Current RS classification includes reactive oxygen species (ROS), reactive nitrogen species (RNS), and reactive sulfur species (RSS), whereof the last two named groups also involve both gasotransmitters, NO and H_2_S. As an example, in a recent study by Zhang et al. [21], the negative impact of elevated concentrations of H_2_S on boar sperm motility was observed. Moreover, the presence of oxidative stress can potentiate the negative impact of high gasotransmitter levels, such as in the case of NO [6], which forms peroxynitrite that inhibits mitochondrial activity [22], reduces sperm motility [23], and increases lipid peroxidation of boar sperm membrane [24]. Thus, the effect of the gasotransmitter on sperm functionality and structural integrity depends not only on its concentration, but also on the specific cellular microenvironment and the presence of other RS. Notwithstanding the recent advances in the study of gasotransmitters and male germ cells under oxidative stress, many questions about the complex interactions and relations remain unanswered.

Most of the information available about these two gasotransmitters and their involvement in the cellular microenvironment originates from the study of somatic cells [25,26,27]. In murine myocardial cells, the application of H_2_S leads to the activation of eNOS, an enzyme responsible for NO production [28], which is also present in boar sperm cells, influencing its function [29]. Both NO and H_2_S share signaling targets, such as the MAPK pathway [13,30], which participates in sperm capacitation [31]. Other common signaling targets represent ion channels (Ca^2+^, K^+^) involved in crucial sperm processes, starting with sperm maturation and ending with oocyte fertilization [32]. Lastly, there seems to be important direct interaction between NO and H_2_S, which contributes to the formation of other signaling molecules with increased signaling potential, such as the nitroxyl radical (HNO) [33]. Among the metabolites resulting from NO and H_2_S interaction, such as nitrosothiols and thionitrous acid [33], the HNO stands out as a potentially relevant molecule for sperm biology concerning oxidative stress [5]. Nitroxyl is formed within seconds after the mixture of NO and H_2_S donors [34] and seems to mimic the action of NO [35]. Moreover, HNO has been proven to also act as an antioxidant agent with the ability to reduce lipid peroxidation of the plasma membrane in the yeast model [36]. Concerning the signaling targets of each gasotransmitter, the most recent experiments dedicated to somatic cells provide solid evidence for the interaction between NO and H_2_S [37,38], yet this phenomenon was not studied in sperm cells to date. Recently, we reviewed [5] the complex interaction of these two gasotransmitters in somatic cells and the potential implications for sperm cell biology. Based on the growing evidence of the interaction between NO and H_2_S in somatic cells, we decided to test the hypothesis of whether the simultaneous application of NO and H_2_S donors can potentiate the effect compared to the single-donor application in boar sperm cells exposed to oxidative stress.

## 2. Materials and Methods

Reagents were purchased from Sigma-Aldrich (Prague, Czech Republic) unless otherwise indicated.

### 2.1. Sample Collection and Experimental Design

Artificial insemination doses from 18 boars of different breeds were purchased from a pig breeding company. Sperm-rich fractions were collected by the gloved-hand method, diluted with Solusem^®^ extender (AIM Worldwide, Vught, The Netherlands; pH ≈ 7), and transported to the laboratory at 17 °C.

Firstly, the morphology was assessed in the suspension of PBS with glutaraldehyde at 2.5% (*v*/*v*) concentration, and only samples with morphologically normal sperm (>75%) were used for the experiment. Sperm samples from three boars were pooled to reduce the effect of male variability and were centrifuged at 167× *g* for 3 min at 17 °C to remove debris and dead sperm cells. The sperm concentration was then checked by using a Bürker chamber, adjusted to 20 × 10^6^ spermatozoa/mL with Solusem^®^.

Sperm samples were then randomly split into five microcentrifuge tubes (certified free of DNA, DNase, RNase, and endotoxins (pyrogens); material: virgin polypropylene; volume: 2 mL; Neptune Scientific, San Diego, CA, USA): Ctr (control sample without oxidative stress) and CtrOX (control sample under oxidative stress). The remaining three tubes were submitted to oxidative stress: NO donor (SNP 100 nM), H_2_S donor (NaHS 100 nM), and their combination (SNP + NaHS 100 nM; duo-donor = DD). All the chemical supplements were diluted in PBS and freshly prepared before the start of each replicate and exposed to light. Oxidative stress was induced by adding a solution of 0.05 mM FeSO_4_ and 0.5 mM sodium ascorbate (Fe^2+^/ascorbate) to the sperm samples. The donors were added to the samples at first and, after approximately 3 min, the oxidative stress was then induced in all samples except Ctr. Sperm analyses were performed after 20 min of incubation for the Ctr sample only and after 90 min of incubation at 38 °C in a water bath for all samples. The experiment was replicated six times with six independent semen pools.

Sperm motility was evaluated using CASA (NIS-Elements; Nikon, Tokyo, Japan, and Laboratory Imaging, Prague, Czech Republic). A prewarmed (38 °C) Spermtrack chamber (PROiSER R + D S.L., Paterna, Spain; chamber depth: 20 µm) was loaded with 5 µL of a sample. A total of 10 sperm kinetic parameters were obtained by analyzing six random fields: total motility (TMot, %), progressive motility (PMot, %), average path velocity (VAP, µm/s), curvilinear velocity (VCL, µm/s), straight-line velocity (VSL, µm/s), the amplitude of lateral head displacement (ALH, μm), beat-cross frequency (BCF, Hz), linearity (LIN, VSL/VCL, %), straightness (STR, VSL/VAP, %), and wobble (WOB, VAP/VCL, %). The settings parameters were as follows: frames per second, 60; minimum frames acquired per sperm track, 31; VAP ≥ 10 μm/s to classify a spermatozoon as motile, STR ≥ 80% to classify a spermatozoon as progressive. A minimum of 200 sperm cells were analyzed for each sample. Sperm motility subpopulations were determined by cluster analysis (see statistical analysis) at 90 min of incubation.

The sperm plasma membrane integrity (PMI) was evaluated as previously described [39]. Aliquots of sperm samples were incubated with carboxyfluorescein diacetate (0.46 mg/mL, *w*/*v*, in dimethyl sulfoxide; DMSO), propidium iodide (0.5 mg/mL, *w*/*v*, in phosphate-buffered saline solution; PBS), and formaldehyde solution (0.3%, *v*/*v*) for 10 min at 38 °C in the dark. Then, 200 spermatozoa were evaluated by using epifluorescence microscopy (40× objective). The spermatozoa showing green fluorescence over the entire head area were considered to have intact plasma membrane.

Acrosome loss was evaluated according to the protocol previously described [40]. After methanol fixation and double washing with PBS, the samples were incubated with peanut agglutinin–fluorescein isothiocyanate (PNA-FITC; 100 µg/mL, *w*/*v*, in PBS) for 10 min at 38 °C in the dark. Epifluorescence microscopy (40× objective) was used to evaluate 200 spermatozoa, and the cells showing no fluorescence over the acrosome were considered as acrosome-lost spermatozoa.

Lipid peroxidation was assessed with the thiobarbituric acid reactive substances (TBARS) assay, as previously described [41]. At the end of each incubation period, sperm aliquots were collected and stored at −80 °C until analysis. The absorbance of each sample was then measured by spectrophotometry at 532 nm (Libra S22, Biochrom, Harvard Bioscience Company, Cambourne, UK). A standard curve was established by using known concentrations of 1,1,3,3-tetramethoxypropane (MDA). The levels of lipid peroxidation are shown as µmol of MDA per 10^8^ spermatozoa. The assay was run in duplicate for each sample. The total antioxidant capacity was determined by spectrophotometry (Libra S22, Biochrom, Harvard Bioscience Company, Cambourne, UK) at 660 nm by using the method described previously [42]. The principle of this assay is based on the antioxidant’s capacity to reduce 2,2′-azino-bis (3-et hylbenz-thiazoline-6-sulfonic acid) (ABTS) previously oxidized with H_2_O_2_. A standard curve was established by using known concentrations of 6-hydroxy-2,5,7,8-tetramethylchroman-2-carboxylic acid (Trolox). The total antioxidant capacity (TAC) was expressed as Trolox equivalents (mM). The assay was run in duplicate for each sample.

### 2.2. Statistical Analysis

Data were analyzed with the statistical program SPSS, version 20 (IBM Inc., Chicago, IL, USA). The generalized linear model (GZLM) was applied to analyze the effects of the NO or H_2_S donor and their combination (DD) on the sperm variables. The statistical significance was determined at *p* < 0.05. Data are shown as the mean ± standard deviation. For determining sperm motility subpopulations, we used two kinetic parameters that de-fine sperm average velocity (i.e., VAP) and trajectory linearity (i.e., LIN). The number of clusters was automatically determined by the two-step cluster component using the Euclidean distance measure and Schwarz’s Bayesian criterion (BIC). After that, the number of clusters previously obtained was used to set up the K-means cluster analysis by using the iteration and classification method. The Kruskal–Wallis analysis was applied to check for differences in sperm subpopulations among treatments. The Wilcoxon signed-rank test (matched samples) was performed to check the differences between kinetic parameters of the subpopulations.

## 3. Results

### 3.1. Sperm Motility

**Figure 1:**
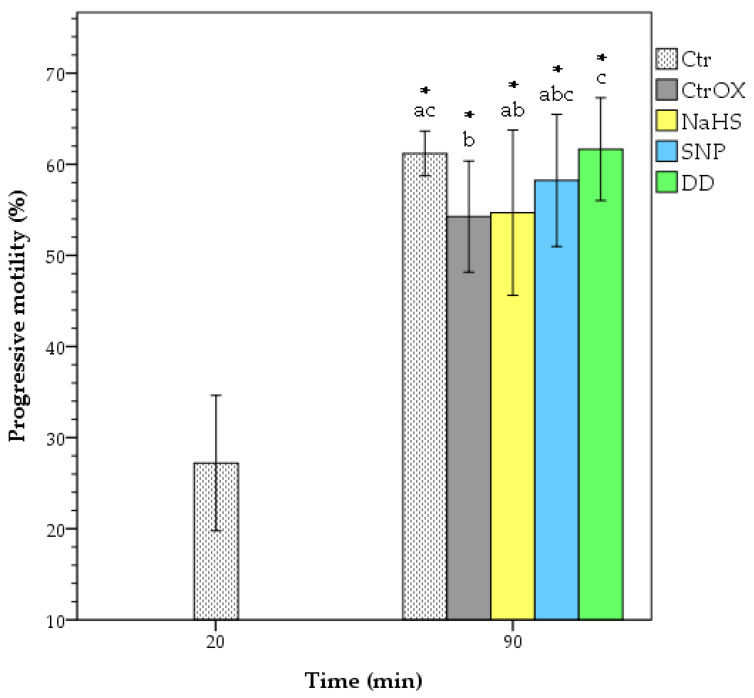
Progressive motility of boar spermatozoa under oxidative stress. Spermatozoa with straightness ≥ 80% were selected as progressive. Different letters indicate significant differences (*p* < 0.05) among treatments at 90 min of incubation. The asterisks indicate significant differences (*p* < 0.05) of samples compared to Ctr at 20 min of incubation. CTR = control; CtrOX = control under oxidative stress; NaHS = 100 nM; SNP = 100 nM; DD = SNP 100 nM + NaHS 100 nM. Data are shown as mean ± SD of 6 replicates.

The PMot of CtrOX was significantly decreased compared to the control sample without oxidative stress (*p* = 0.048), as seen in Figure 1. The PMot of DD treatment (61.7%) was the only one that significantly exceeded the PMot of the CtrOX sample (54.3%; *p* < 0.05). Moreover, it was also significantly higher than the PMot of NaHS treatment (54.7%; *p* < 0.05). The NaHS had a tendency of lower PMot than the Ctr sample (61.2%; *p* = 0.064). SNP was the only treatment that did not statistically differ from any other sample at 90 min of incubation time. During the incubation of all samples, the value of VSL, LIN, and STR increased, which resulted in more rectilinear trajectories compared to the Ctr sample at 20 min of incubation.

The CtrOX had significantly reduced total motility (TMot) compared to Ctr sample (*p* = 0.038). In continuation, all donor samples under oxidative stress had comparable TMot to control sample without oxidative stress (*p* > 0.05), although TMot of NaHS treatment tended to be lower than the in Ctr sample (*p* = 0.058). A significant difference (*p* < 0.05) among treatments was seen between BCF of DD treatment, which was higher than the one of NaHS. Interestingly, the BCF of the DD sample tended to be higher than the Ctr sample (*p* = 0.076). Complete kinetic parameters are shown in Table 1. Cluster analysis rendered two sperm subpopulations that, based on their kinetics, were classified as rapid progressive (Sp1) and slow nonprogressive (Sp2). Sperm subpopulations differed among them in all of the sperm kinetic parameters (Table S1). However, there were no significant differences between treatments (*p* > 0.05; Figure S1). Interestingly, NaHS showed the smallest Sp1 (38.2%), which was 1% smaller than Sp1 of CtrOX (39.2%). SNP treatment contained 42.9% of rapid progressive spermatozoa (Sp1). Yet, the Sp1 was most represented in the DD sample (44.6%) that also exceeded the Sp1 of Ctr (43.8%; *p* > 0.05).

### 3.2. Plasma Membrane Integrity and Lipid Peroxidation

**Figure 2:**
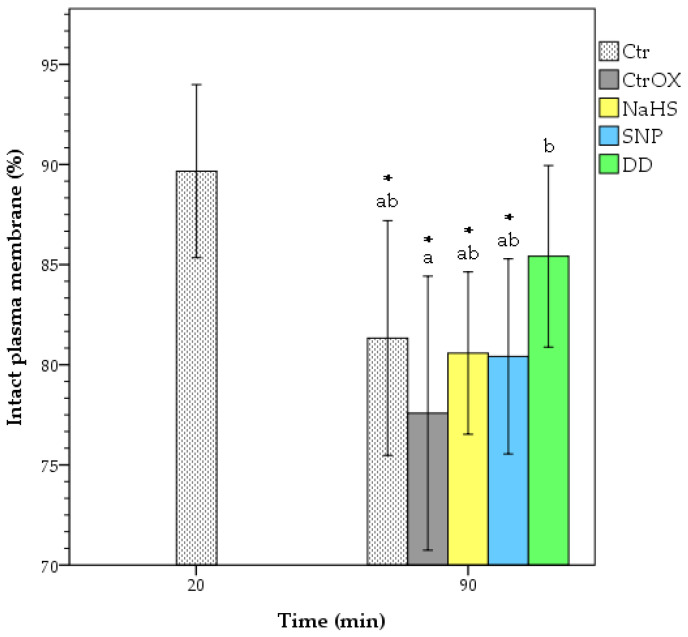
Percentage of membrane intact boar spermatozoa under oxidative stress. Different letters indicate significant differences (*p* < 0.05) between treatments at 90 min of incubation. The asterisks indicate significant differences (*p* < 0.05) of samples compared to Ctr at 20 min incubation. CTR = control; CtrOX = control under oxidative stress; NaHS = 100 nM; SNP = 100 nM; DD = SNP 100 nM + NaHS 100 nM. Data are shown as mean ± SD of 6 replicates.

**Figure 3:**
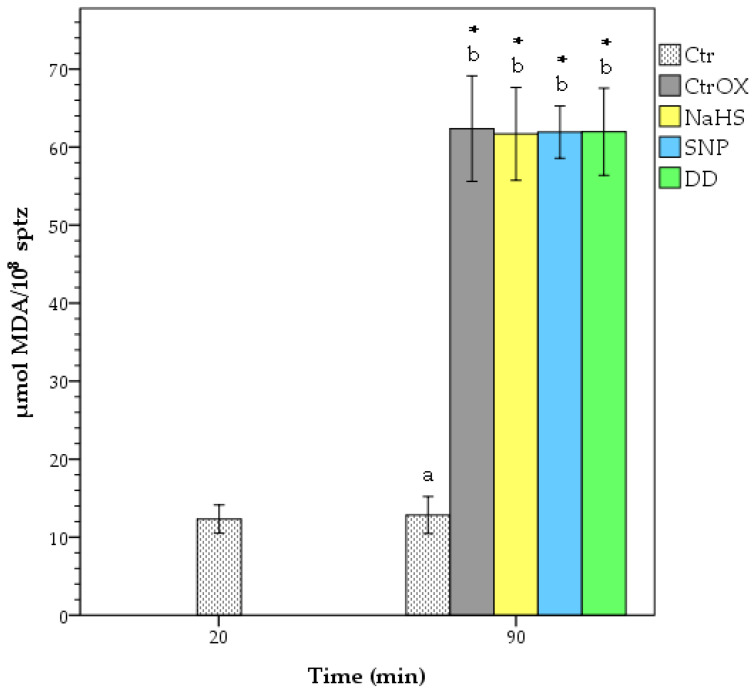
Levels of lipid peroxidation of boar spermatozoa under oxidative stress. Different letters indicate significant differences (*p* < 0.05) between treatments at 90 min of incubation. The asterisks indicate significant differences (*p* < 0.05) of samples compared to Ctr at 20 min incubation. CTR = control; CtrOX = control under oxidative stress; NaHS = 100 nM; SNP = 100 nM; DD = SNP 100 nM + NaHS 100 nM. Data are shown as mean ± SD of 6 replicates.

There was no difference in PMI between Ctr and CtrOX at 90 min of incubation time (Figure 2). The treatment DD showed a higher PMI in comparison to the CtrOX (*p* < 0.05). Although not significant (*p* > 0.05), only in DD treatment there was a higher PMI percentage than in the Ctr sample at 90 min of incubation. Moreover, DD treatment was the only one that did not show significant differences (*p* > 0.05) with the Ctr sample at 20 min of incubation. Yet, there was a decrease in PMI of the Ctr sample during incubation (89.7% vs. 81.3% respectively; *p* < 0.05). Moreover, there was a tendency in DD treatment to have higher PMI than treatments with NaHS and SNP alone (*p* = 0.076 and *p* = 0.067 respectively). All samples under oxidative stress showed higher levels of lipid peroxidation (LP; *p* < 0.05) than the control sample without oxidative stress (Figure 3). No significant differences (*p* > 0.05) among treatments under oxidative stress were found in terms of LP. The levels of LP in the Ctr sample did not change during incubation time (*p* > 0.05).

### 3.3. Acrosome Integrity

No significant differences were found among the samples. Interestingly, the treatment SNP had the lowest percentage of intact acrosome and tended to be lower than the Ctr sample (*p* = 0.085), and then DD treatment (*p* = 0.075). Moreover, the SNP treatment had the highest standard deviation, which was more than 3 times higher than in any other treatment (Table 2).

### 3.4. Total Antioxidant Capacity

As shown in Table 3, the TAC was significantly reduced in all treatments under oxidative stress compared to the Ctr sample at 90 min of incubation (*p* < 0.05). No significant differences were observed between the treatments under oxidative stress. TAC of the Ctr sample without oxidative stress decreased during the incubation (*p* < 0.05).

## 4. Discussion

In the present study, we investigated the differences in boar sperm quality traits in samples under oxidative stress supplemented with NO donor (SNP 100 nM), H_2_S donor (NaHS 100 nM), and their combination (SNP + NaHS 100 nM; DD). Our results support the hypothesis that there is a synergy between NO and H_2_S protecting membrane integrity and increasing progressive motility in boar spermatozoa under oxidative stress. This is the first study performed on male gametes testing the simultaneous NO and H_2_S donor application. Several combinations of NO and H_2_S donors have been tested in somatic cells to date and this is the first study that has tested the combination of NO/H_2_S donors on sperm cells to the best of our knowledge. In extensive research by Yong et al. [43,44], the combination of SNP and NaHS was tested in the cardiovascular system. In the study from 2010 [43], the author collective established the ideal ratio between the two above-mentioned donors to be 1:1 in terms of the effectiveness of cardiomyocyte shortening. Moreover, Yong et al. [43] indirectly demonstrated that HNO is formed as the result of the two donors’ combination using HNO scavengers. NaHS together with Na_2_S is the most common sulfide salt used in a biological system as H_2_S donors. The two sulfide salts can substitute each other in terms of main characteristics, both being fast and direct donors releasing relatively high amounts of H_2_S in a short period of time [45]. In a study from 2014, Eberhardt et al. [34] tested the combination of NO and H_2_S donors (DEA NONOate, Na_2_S, respectively) in the neurovascular system. Using an HNO-selective electrode, they have demonstrated that the mixture of NO and H_2_S (Na_2_S donor) leads to immediate HNO formation. The peak of HNO formation was observed after 1 min of the NO and Na_2_S mixture [34]. Based upon these studies and our preliminary experiments, we decided to use the combination of two fast-releasing NO and H_2_S donors, SNP and NaHS, and established the ratio and concentration at 100 nM:100 nM. The concentration of each donor used was also set considering our previous studies [4,46] that indicated the effective concentrations of each NO and H_2_S donor in boar spermatozoa. Moreover, we bore in mind the estimated physiological concentrations, which, in general, are within the nM range for both gasotransmitters [47]. To artificially induce oxidative stress, we used Fe^2+^/ascorbate as a ROS-generating system. This model is suitable for studies of lipid peroxidation [41], which belongs to the main causes of sperm membrane degradation [48]. The induction of oxidative stress in our study results in decreased motility (TMot and PMot) and TAC, and increased LP in the CtrOX sample compared to Ctr. Comparing the treatments containing donors alone and their combination, some interesting differences were observed in sperm motility and PMI.

The DD treatment with two donors combined show significantly higher PMI and PMot than CtrOX, and also preserved PMI compared to Ctr at 20 min. Moreover, DD treatment exceeded NaHS treatment in terms of PMot. Interestingly, SNP treatment exerted a mild effect with TMot and PMot values in between the ones of NaHS and DD, respectively. Moreover, SNP was characterized by a higher standard deviation in the case of acrosome integrity compared to the rest of the treatments. Perhaps the formation of more reactive and pro-oxidative molecules, such as peroxynitrite, could explain the reduced positive effect on plasma membrane integrity and sperm motility, and also the partial acrosome destabilization when compared to DD treatment. On the other hand, NaHS treatment showed no effect and tended to have several lower sperm quality traits (e.g., TM) in comparison to Ctr. We speculate that, in such a low dose, only a partial increase in activity of SOD occurred, depleting the H_2_S pool. This could result in mild protection of plasma membrane integrity compared to CtrOX, although no increase in sperm motility was seen. Taken together, these results indicate that the two donors combined more resemble the action of SNP alone rather than NaHS in the presence of oxidative stress. Yet, the combination of both donors seems to be more efficient than SNP alone, as seen in the case of progressive motility. This leads to speculation that the interaction of the two donors results in the formation of a more potent metabolite that mimics the action of NO (Figure S2). Indeed, various studies state that the interaction between H_2_S and NO leads to the formation of HNO, which mimics the action of NO [33,49]. HNO resulting from NaHS and NO interaction seems to be a more potent signal transductor than its precursor, NO, in the vascular system [33]. Yet, this hypothesis remains to be tested. In a previous study performed on human spermatozoa [13], NaHS donor used at 5 µM concentration under in vitro conditions impaired sperm motility. Accordingly, in our previous study [4], Na_2_S donor had a positive effect at the lowest concentration (3 µM) on TMot but impaired the quality of boar spermatozoa at higher concentrations. In this study, NaHS did not affect boar spermatozoa at 100 nM concentration. These results indicate that the doses with a positive effect on sperm quality are at very low micromolar concentrations in the case of fast-releasing donors of H_2_S applied to boar spermatozoa in vitro and under oxidative stress. Overall, we observed higher values of various sperm quality traits (TMot, PMot, PMI) in samples treated with SNP 100 nM compared to the control sample with oxidative stress, although not statistically significant. In another study performed by Hellstorm et al. [12], thawed human spermatozoa were treated with SNP at 50 and 100 nM concentration, and positive effects on sperm viability (eosin staining) and motility and reduction in lipid peroxidation were observed. The difference in the results could be attributed to the fact that, in our study, the positive effect might be masked by the presence of additionally induced oxidative stress. The reaction of NO with ROS results in the formation of peroxynitrite, which impairs sperm quality traits and the state of lipid peroxidation in boar spermatozoa at micromolar concentrations [24]. An interesting observation was made concerning the kinetic parameters of the CASA analysis. The sample DD had a significantly higher value of beat-cross frequency (BCF) than the sample NaHS, indicating a difference in motility pattern. The BCF parameter is suggested as one of the predictive factors of boar fertility and insemination success [50].

As stated above, an increased PMI was observed in the treatment containing both donors (DD), which was the only one that significantly exceeded the PMI of the CtrOX sample. Moreover, the DD treatment did not differ from the control sample without oxidative stress at 20 min of incubation. The percentage of spermatozoa with an intact membrane is associated with increased fertilization potential in boar [51,52,53], and other mammals as well [54,55]. Similarly to the plasma membrane, acrosome integrity is essential for the proper function of a sperm cell during fertilization in mammals [56]. Despite no statistical significance, an interesting observation concerning acrosome integrity of SNP treatment was made, since the standard deviation was more than doubled in comparison to all other treatments. Numerous studies dedicated to the effect of NO on spermatozoa demonstrated its involvement in acrosomal reaction [1,57,58]. The SNP at µM concentrations triggers acrosome reaction in human spermatozoa [59,60] and boar spermatozoa [61,62]. Our results indicate the possibility that partial acrosome membrane destabilization occurred in samples treated with 100 nM SNP. Perhaps this occurred due to conversion of NO in the presence of oxidative stress to ONOO^−^, which is a potent acrosomal reaction inducer.

## 5. Conclusions

For the first time, the effect of simultaneous application of NO and H_2_S donors has been tested in boar spermatozoa exposed to oxidative stress. Our results indicate a possible synergy between the two gasotransmitters, increasing progressive motility and protecting plasma membrane integrity. The dual NO and H_2_S donor application was the only one that resulted in an increased PMot and PMI compared to CtrOX. Interestingly, SNP treatment was rather similar to DD. On the other hand, NaHS treatment showed impaired PMot compared to DD and converged to lower motility (TMot and PMot) than Ctr. These results indicate the importance and the complexity of gasotransmitter interactions in the male gametes.

## Figures and Tables

**Table 1:** The effect of NaHS, SNP, and their combination (DD) on kinetic parameters of boar spermatozoa under oxidative stress.

Treatment	Time (min)	TMot (%)	VAP (µm/s)	VCL (µm/s)	VSL (µm/s)	ALH (µm)	BCF (Hz)	LIN (%)	STR (%)	WOB (%)
Ctr	20	65.9 ± 8.2	40.1 ± 6.1	95.2 ± 8.2	24.9 ± 4.7	3.0 ± 0.3	11.4 ± 0.7	28.4 ± 4.7	66.6 ± 6.0	40.8 ± 3.6
Ctr	90	66.8 ± 2.9 ^a^	40.5 ± 4.3	71.7 ± 6.9 *	36.7 ± 4.1 *	2.8 ± 0.3	14.0 ± 0.5 ^ab^^,^*	50.0 ± 2.8 *	88.4 ± 1.6 *	54.9 ± 2.4 *
CtrOX	90	55.7 ± 11.2 ^b^	41.0 ± 8.4	77.5 ± 17.5 *	34.4 ± 7.1 *	2.7 ± 0.6	14.2 ± 0.8 ^ab^^,^*	48.8 ± 7.6 *	85.4 ± 6.3 *	55.3 ± 5.1 *
NaHS	90	56.7 ± 13.1 ^ab^	38.8 ± 4.8	71.0 ± 13.7 *	33.8 ± 3.7 *	2.6 ± 0.4	13.8 ± 0.5 ^a^^,^*	49.5 ± 8.2 *	86.7 ± 7.9 *	55.2 ± 5.0 *
SNP	90	59.4 ± 12.2 ^ab^	40.9 ± 5.9	72.2 ± 11.3 *	36.4 ± 4.8 *	2.7 ± 0.4	14.3 ± 1.0 ^ab^^,^*	51.9 ± 5.5 *	88.7 ± 4.4 *	57.1 ± 4.3 *
DD	90	60.5 ± 9.8 ^ab^	40.2 ± 6.1	70.0 ± 15.5 *	36.2 ± 4.8 *	2.7 ± 0.5	14.7 ± 1.1 ^b^^,^*	53.4 ± 7.2 *	89.7 ± 5.6 *	58.2 ± 5.0 *

Different letters indicate significant differences (*p* < 0.05) among treatments at 90 min of incubation. The asterisks indicate significant differences (*p* < 0.05) of samples compared to Ctr at 20 min incubation. CTR = control; CtrOX = control under oxidative stress; NaHS 100 nM; SNP = 100 nM; DD = SNP 100 nM + NaHS 100 nM. TMot: total motility; VAP: average path velocity; VCL: curvilinear velocity; VSL: straight-line velocity; ALH: amplitude of lateral head displacement; BCF: beat-cross frequency; LIN: linearity (VSL/VCL); STR: straightness (VSL/VAP); WOB: wobble (VAP/VCL). Data are shown as the mean ± SD of 6 replicates.

**Table 2:** The effect of NaHS, SNP, and their combination (DD) on percentage of acrosome intact boar spermatozoa under oxidative stress.

Treatment	Time (min)	Intact Acrosome (%)
Ctr	20	98.0 ± 1.4
Ctr	90	97.8 ± 2.0
CtrOX	90	96.2 ± 1.2
NaHS	90	97.1 ± 1.6
SNP	90	95.5 ± 5.3
DD	90	97.9 ± 1.4

CTR = control; CtrOX = control under oxidative stress; NaHS = 100 nM; SNP = 100 nM; DD = SNP 100 nM + NaHS 100 nM. Data are shown as mean ± SD of 6 replicates.

**Table 3:** The effect of NaHS, SNP, and their combination (DD) on the levels of total antioxidant capacity in different samples of boar spermatozoa under oxidative stress.

Treatment	Time (min)	Total Antioxidant Capacity (mM)
Ctr	20	0.60 ± 0.06
Ctr	90	* 0.54 ± 0.06 ^a^
CtrOX	90	* 0.43 ± 0.04 ^b^
NaHS	90	* 0.42 ± 0.04 ^b^
SNP	90	* 0.41 ± 0.04 ^b^
DD	90	* 0.42 ± 0.05 ^b^

Different letters indicate significant differences (*p* < 0.05) among treatments at 90 min of incubation. The asterisks indicate significant differences (*p* < 0.05) of samples compared to Ctr at 20 min incubation. CTR = control; CtrOX = control under oxidative stress; NaHS = 100 nM; SNP = 100 nM; DD = SNP 100 nM + NaHS 100 nM. Data are shown as mean ± SD of 6 replicates.

## Data Availability

The data presented in this study are available in Supplementary Materials (Dataset S1 and S2).

## Supplementary Materials

The following supporting information can be downloaded at: https://www.mdpi.com/article/10.3390/ani12050602/s1, Figure S1: Distribution of subpopulations among treatments at 90 min of incubation; Figure S2. The physiological action of NO and H_2_S and the hypothesized action related to observed results; Table S1. Kinetic parameters of different subpopulations
