# Supplementary material for: The Interaction of NO and H2S in Boar Spermatozoa under Oxidative Stress
Source: Animals (Basel). 2022 Feb 28;12(5):602. doi: 10.3390/ani12050602 (PMC8909797; doi:10.3390/ani12050602)
Supplement: Supplementary file 1 [file animals-12-00602-s001.zip › SF1_SF2_ST1.pdf]

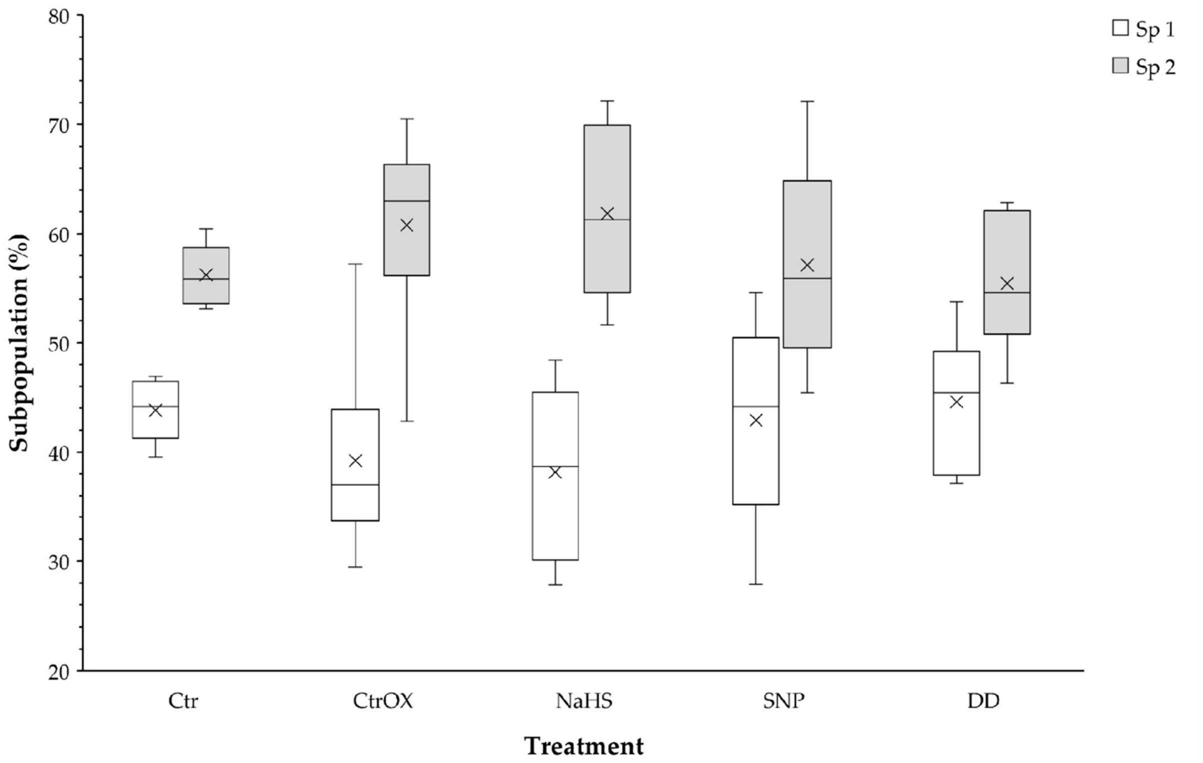

Figure S1. Distribution of subpopulations among treatments at 90 min of incubation. Sp1: rapid progressive; Sp2: slow non-progressive. There were no differences among boar spermatozoa samples within each sperm subpopulation. The cross indicates the mean of a given subpopulation.

Table S1. Kinetic parameters of different subpopulations.

Parameter	Sp1	Sp2
VAP (μm/s)	49.8 ± 7.7[a]	9.3 ± 2.3[b]
VCL (μm/s)	78.8 ± 16.0[a]	35.4 ± 9.0[b]
VSL (μm/s)	44.7 ± 6.3[a]	6.5 ± 1.4[b]
ALH (μm)	3.1 ± 0.5[a]	1.1 ± 0.2[b]
BCF (Hz)	16.2 ± 0.9[a]	7.4 ± 0.4[b]
LIN (%)	60.0 ± 6.2[a]	19.4 ± 1.3[b]
STR (%)	91.4 ± 5.3[a]	61.3 ± 2.4[b]
WOB (%)	64.9 ± 4.0[a]	27.6 ± 1.3[b]

Sp1: rapid progressive; Sp2: slow non-progressive. CTR = control; CtrOX = control under oxidative stress; NaHS 100 nM; SNP = 100 nM; DD = SNP 100 nM + NaHS 100 nM. TMot: total motility; VAP: average path velocity; VCL: curvilinear velocity; VSL: straight-line velocity; ALH: amplitude of lateral head displacement; BCF: beat-cross frequency; LIN: linearity (VSL/VCL); STR: straightness (VSL/VAP); WOB: wobble (VAP/VCL). Different superscripts within the same row indicate significant differences ($p < 0.05$). Data are shown as mean ± SD.

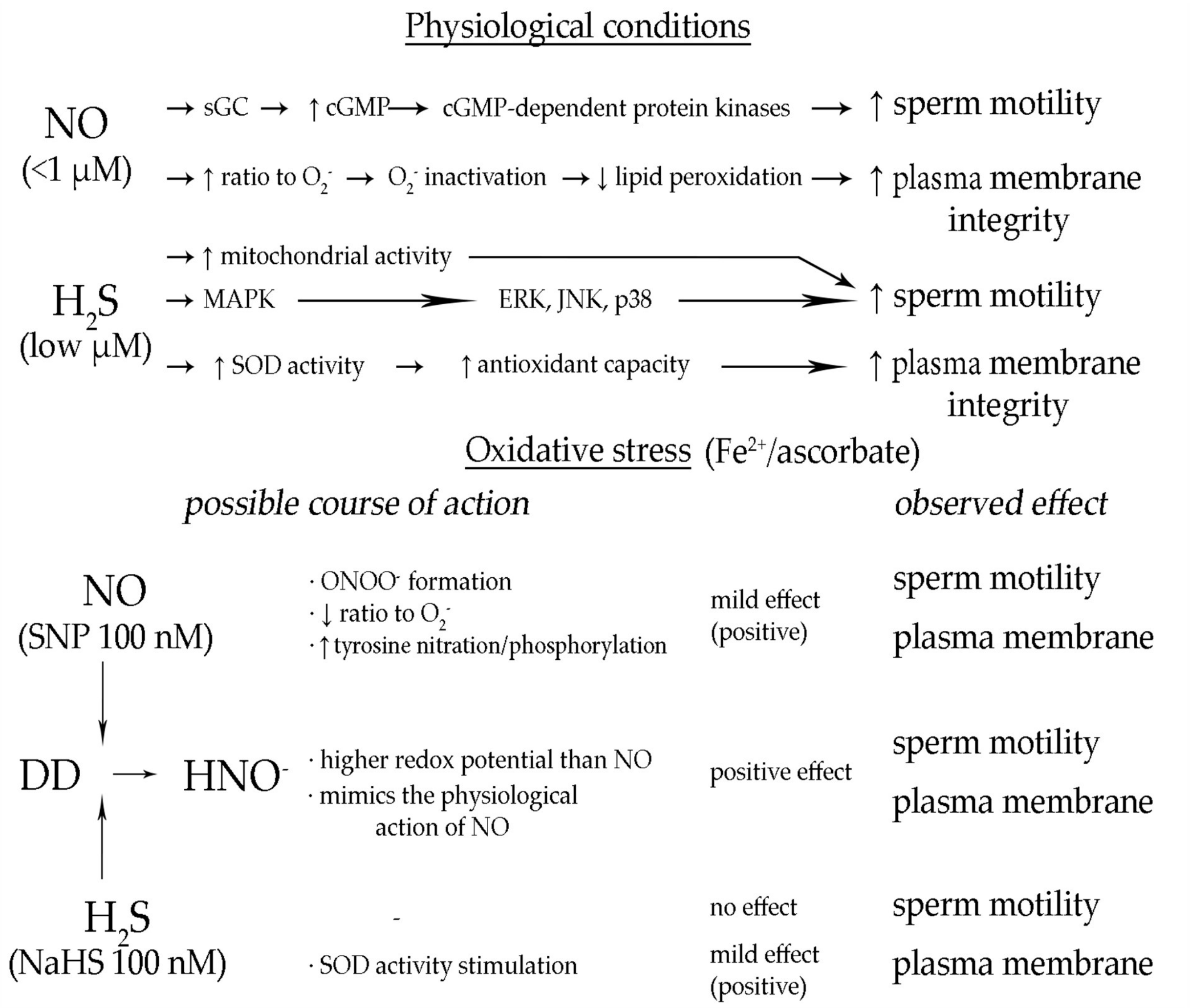

Figure S2. The physiological action of NO and H_2S and the hypothesized action related to observed results. The chart describes the involvement of NO and H_2S in the most relevant parameters (sperm motility and plasma membrane integrity) to the results. Physiologically, NO promotes sperm motility mainly through activation of sGC and can reduce lipid peroxidation by decreasing its ratio to superoxide. On the other hand, H_2S is known to affect the mitochondrial activity and activate the MAPK pathway which results in increased sperm motility. Moreover, it also increases the SOD activity and thus protects the cell from oxidative stress. In the experiment, we hypothesize that the NO alone in the presence of oxidative stress may result in the formation of more reactive molecules that would explain observed effects on sperm motility and plasma membrane integrity. Also, some of the metabolites such as $ONOO^-$ are known to be effective inducers of acrosomal reaction. H_2S at such low dosis could potentially just slightly up-control the SOD activity protecting the plasma membrane integrity with no other effect due to its depletion. We hypothesize that HNO is formed upon the interaction of the two gasotransmitters. HNO is known to be a stronger signal transducer and exerts antioxidant properties through the reduction of lipid peroxidation. cGMP, cyclic guanosine monophosphate; ERK, extracellular signal-regulated kinase; JNK, C-Jun N-terminal kinase; MAPK, mitogen-activated protein kinases; sGC, soluble guanylyl cyclase; SOD, superoxide dismutase.
