# Supplementary figures and images for: The Interaction of NO and H2S in Boar Spermatozoa under Oxidative Stress
Source: Animals (Basel). 2022 Feb 28;12(5):602. doi: 10.3390/ani12050602 (PMC8909797; doi:10.3390/ani12050602)

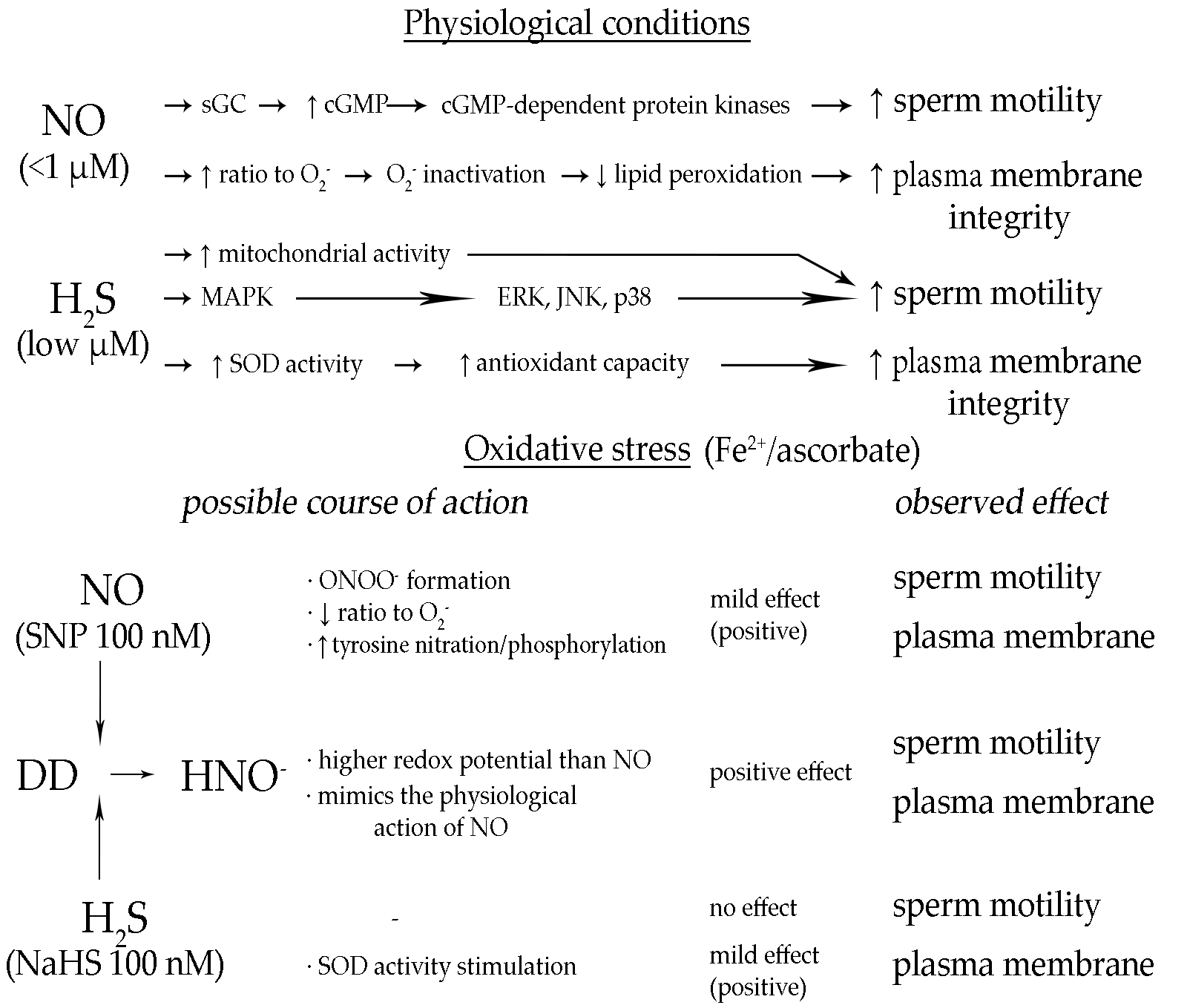

Supplement: Supplementary file 1 [file animals-12-00602-s001.zip › Figure S2.png]
